# Eight enteric-coated 50 mg diclofenac sodium tablet formulations marketed in Saudi Arabia: in vitro quality evaluation

**DOI:** 10.1186/s13104-020-05270-4

**Published:** 2020-09-14

**Authors:** Muhammad M. Hammami, Rajaa F. Hussein, Reem AlSwayeh, Syed N. Alvi

**Affiliations:** 1 Clinical Studies and Empirical Ethics Department, King Faisal Specialist Hospital and Research Centre, MBC 03, PO Box # 3354, Riyadh, 11211 Saudi Arabia; 2 Alfaisal University College of Medicine, Riyadh, Saudi Arabia

**Keywords:** Diclofenac sodium tablet, In-vitro quality, Saudi Arabia, Dissolution profile, Immediate-release, Generic formulations

## Abstract

**Objective:**

To evaluate in vitro quality of enteric-coated 50 mg diclofenac sodium tablet formulations on Saudi market.

**Results:**

A reference and seven generic (G1-7) formulations were commercially available in December 2019/January 2020 and were assessed within 25–75% of manufacture-expiration period. Weight variation (range as% difference from mean, n = 20), active substance content (ASC, mean (SD) as% difference from label, n = 20), hardness (mean (SD), n = 10), and friability (% weight loss, n = 20) were 97–103%, 102.0% (3.4%), 15.4 (1.1) kg, and 0.24%, respectively, for the reference. For G2-7, they were ≤ ±5%, 98.6% (4.0%) to 109.9% (1.8%), 11.9 (0.9) to 18.3 (0.8) kg, and ≤ 0.00 to 0.75%, respectively. G1 ASC, hardness, and friability were 111.3% (1.7%), 20.1 (1.7) kg, and 1.10%, respectively. Disintegration time (n = 6) and dissolution profile (n = 8) were also determined. No formulation disintegrated or released ˃ 0.1% of label ASC in 0.1 N HCl for 2 h. The reference disintegrated in 15:00 min:seconds and released a mean (range) of 100% (99–103%) of label ASC by 45 min in phosphate buffer (pH = 6.8). G1-7 disintegrated in 8:53 to 20:37 min:seconds and released 81% (69–90%) (G1) to 109%. Except for borderline performance of G1, all formulations passed in vitro quality tests according to United States Pharmacopoeia.

## Introduction

Quality generic drug products save money and improve accessibility to health care [1]. However, lay people [2] and healthcare workers [3] not infrequently question the quality of marketed generic drug products, resulting in their suboptimal use. Although generic products must pass bioequivalence testing before entering the Saudi market [4], ongoing assessment is essential to ensure that the desired quality is maintained post-marketing.

We have conducted pre-marketing [5–12] and post-marketing [13] bioequivalence studies of several drug products in Saudi Arabia. However, increasing emphasis is being placed on in vitro physiochemical quality testing [14–16] because it does not involve human subjects and have lower cost.

Diclofenac (as sodium or potassium salt) belongs to the non-steroidal anti-inflammatory drug group [17] and is one of the widely manufactured and marketed drugs in Saudi Arabia; more than 30 formulations are listed on the Saudi formulary [4]. Further, three of diclofenac products were among the top four drugs sold in Saudi Arabia in the period of 2010 to 2015 [18].

Few studies have examined in vitro quality of marketed diclofenac sodium formulations [19–23]. Some did not include a reference formulation [22, 23], two identified inferior products [19, 21], all used ultraviolet spectrophotometer for drug level analysis rather than high performance liquid chromatography (HPLC), and none included products from the Saudi market.

The aim of this study was to evaluate in vitro quality of enteric-coated 50 mg diclofenac sodium tablet formulations that are commercially available on the Saudi market.

## Main text

### Drugs and chemicals

All single-drug tablet formulations of 50 mg diclofenac sodium that were commercially available in retail pharmacies in Riyadh, Saudi Arabia during the period December 2019/January 2020 and were within 25%–75% of their manufacture-expiration period were included in the study. Label information of the included formulations (a reference and seven generic (G1 to G7) formulations) is presented in (Additional file 1: Table S1, Label information). Six other generic formulations were listed on the Saudi Formulary [4] but were not commercially available and were not included in the study.

Diclofenac sodium standard was purchased from Sigma-Aldrich (St Louis, MO, USA), HPLC grade acetonitrile and methanol from Fisher Scientific Co. (Loughborough, UK), disodium hydrogen phosphate from Fluka (Buchs, Switzerland), potassium phosphate monobasic and glacial acetic acid from Fisher Chemical (Fair Lawn, New Jersey, USA), and hydrochloric acid (HCl) from Merck (Darmstadt, F.R. Germany).

### Instruments

HPLC-dissolution system (Waters Associates, Inc. Milford, MA. USA) consisted of Waters 2690D Separation Module with eight-needle dissolution dispenser, Waters Transfer Module with eight syringes, one dissolution test bath (Hanson Research SR8-Plus, USP dissolution apparatus II (paddle)), eight Uni-Probes, and Waters 996 Photodiode array detector set at 276 nm. Other instruments used included an electronic balance (Model AG 204, Mettler Toledo, Greifensee, Switzerland), as well as Microprocessor Disintegration Test Apparatus (Model SSE-731), Microprocessor Friability Apparatus (Model SSE-710), and Digital Tablet Hardness Tester (Model SSE-DIGIT AB-SPV), all from Sunshine Scientific Equipments, Delhi, India.

### Sample preparation and HPLC assay

A stock solution of diclofenac sodium (1000 µg/ml) was prepared in methanol and stored at − 20º C. It was diluted in a phosphate buffer (pH 6.8 ± 0.05) composed of 0.05 M disodium hydrogen phosphate and 0.05 M potassium dihydrogen phosphate (50:50, v:v) to produce standard curve (0.1, 0.5, 1.0, 5.0, 10.0, 20.0, 40.0, 60.0 and 80.0 µg/ml) and quality control (1.5, 7.5, 15, and 50 μg/ml) samples. This phosphate buffer was also used for disintegration and dissolution tests and for determining active substance content (ASC). A standard curve and three sets of quality control samples were used in each run. The HPLC assay uses liquid–liquid extraction and naproxen as an internal standard, and is linear (R^2^ ≥ 0.998) in the range 0.1–80.0 µg/ml [24]. It was used to determine ASC in tablets and the dissolution profile. No interference from tablet’s excipients was observed.

### Quality control tests and calculations

Weight variation test: 20 randomly-selected unites of each formulation were examined. Mean (SD) was calculated and% deviation of individual unit weight from mean weight of the formulation was determined.

Friability test: 20 randomly-selected unites of each formulation were examined. They were weighted, placed in the friabilator (25 revolutions/minute for 4 min), de-dusted and weighted again, and friability was determined as% weight loss.

Hardness test: 10 randomly-selected unites of each formulation were examined. Mean (SD) required pressure to break diametrically placed tablets was determined.

ASC test: 20 randomly-selected unites of each formulation were examined. Tablets were individually crushed using morter, dissolved in 100 ml methanol, filtered with a syringe using 0.2 µm filter, diluted with 9.0 ml phosphate buffer, and 100 µl was injected in the HPLC system. Mean (SD) content in mg and percent deviation of individual unites from label were determined.

Disintegration test: 6 randomly-selected unites of each formulation were examined using 0.1 N HCL for 2 h followed by phosphate buffer (pH 6.8) as disintegration medium. The basket rack was placed in a 1000 ml vessel containing 900 ml disintegration medium maintained at 37 ± 2 °C with the test unit remaining 1.5 cm below the surface of the liquid on their upward movement and above 2.5 cm from the bottom of the beaker in their downward movement. The basket rack moved up and down (5–6 cm) at a frequency of 31 cycles per minute. Range of disintegration time (time to no particle on the basket) was determined.

Dissolution test: 8 randomly-selected unites of each formulation were examined using 0.1 N HCL for 2 h followed by phosphate buffer (pH 6.8) as dissolution medium (900 ml), one unite in each vessel, a stirring rate of 50 ± 1 rpm, and a temperature of 37 ± 0.5 °C. The test ended with a stirring rate of 250 rpm for 15 min (infinity). A sample of 1.0 ml was withdrawn from a zone midway between the surface of the dissolution medium and the top of the rotating blade (not less than 1 cm from the vessel wall) and was immediately replaced with an identical volume of fresh medium. Samples were withdrawn at 60 and 120 min in 0.1 N HCl and at 10, 15, 20, 30, 45, 60, 90 and 105 min in phosphate buffer. 100 µl of the 1 ml samples were injected into the HPLC system. The vessel was kept covered for the duration of the test, the temperature of the mixture was verified at suitable times, and the behavior of the unit was observed throughout the dissolution testing. Mean (SD) amount released and% of label ASC released at each time point was determined. Time to release 50% of label ASC was also determined.

## Results

Main results are summarized in the Table 1. Mean weight of the eight formulations ranged from 192.5 (3.5) to 283.6 (4.0) mg. Weight range was 97–103% of mean weight for reference formulation and within ≤ ±5% for all 7 generic formulations.

**Table 1:** In-vitro quality of a reference and seven generic enteric-coated 50 mg diclofenac sodium tablet formulations available on the Saudi market

Code	Weight n = 20		Active substance content^b^ n = 20		Hardness^c^ n = 10	Friability^d^ n = 20	Disintegration^e^ (phosphate buffer) n = 6	Dissolution^f^ (phosphate buffer) n = 8
	Mean (SD) mg	Range^a^ % from mean	Mean (SD) mg	Mean (SD) % of label	Mean (SD) kg	%Loss	Range Minute: second	Mean (range) release at 45 min% of label
R	212.9 (3.6)	97–103	51.0 (1.7)	102.0 (3.4)	15.4 (1.1)	0.24	15:00–15:00	100 (99–103)
G1	203.7 (3.0)	97–102	55.7 (0.9)	111.3 (1.7)	20.1 (1.7)	1.10	14:09–14:09	81(69–90)
G2	208.9 (4.3)	95–103	53.8 (2.0)	107.6 (4.0)	16.6 (1.2)	0.00	17:23–19:19	106 ( 102–109)
G3	192.5 (3.5)	96–103	49.3 (2.0)	98.6 (4.0)	12.8 (0.4)	0.00	13:29–13:29	101 (94–113)
G4	283.6 (4.0)	97–103	50.9 (1.7)	101.9 (3.4)	11.9 (0.9)	0.16	20:37–20:37	108 (102–112)
G5	232.1 (3.3)	98–103	51.7 (2.2)	103.3 (4.4)	16.8 (1.3)	0.00	18:00–18:00	102 (101–103)
G6	243.9 (2.2)	99–103	54.9 (0.9)	109.9 (1.8)	18.3 (0.8)	0.75	9:00–9:00	109 (107–112)
G7	219.9 (2.8)	98–102	52.0 (2.0)	104.0 (4.0)	14.4 (0.7)	0.20	8:53–8:53	104 (100–107)

^a^Acceptable variation limits ≤ ±7.5% for tablets ˃  80 and < 250 mg and ≤ ±5% for tablets ≥ 250 mg; to pass, no more than 2/20 tablet differ by more than the percentage permitted and no one tablet differ by more than double the percentage. ^b^ Acceptable limits, mean content 90–110% of label. ^c^Optimum hardness for coated tablets 10–20 kg. ^d^Acceptable limit ≤ 1%. ^e^None disintegrated in 0.1 N HCl for 2 h. Acceptable limits, no disintegration in 0.1 N HCl for 2 h and complete disintegration in phosphate buffer (pH 6.8) within 60 min. ^f^0 to 0.1% release was observed in 0.1 N HCL for 2 h. Acceptable limits, release of ≤ 10% of label in 0.1 N HCl and ≥ 75 + 5% in phosphate buffer (pH 6.8)

Mean (SD) ASC and percent difference from label (50 mg) for the reference formulation were 51.0 (1.7) mg and 102.0% (3.4%), respectively. All generic formulations but G1 had a mean ASC between 90 and 110% of label. G1 had mean (SD) ASC and percent difference of 55.7 (0.9) mg and 111.3% (1.7%), respectively.

The reference formulation had a mean (SD) hardness of 15.4 (1.1) kg and lost 0.24% of its weight during friability testing. All generic formulations but G1 passed the hardness test (optimum 10–20 kg) and had a friability of ≤ 1%. G1 had borderline hardness and friability of 20.1 (1.7) and 1.10%, respectively.

Although not all formulations were labeled as enteric-coated, none disintegrated in 0.1 N HCl for 2 h. On the other hand, phosphate buffer (pH 6.8) disintegration time of the reference formulation was 15.00 min:seconds; and all generic formulations disintegrated in less than 21 min.

**Fig. 1:**
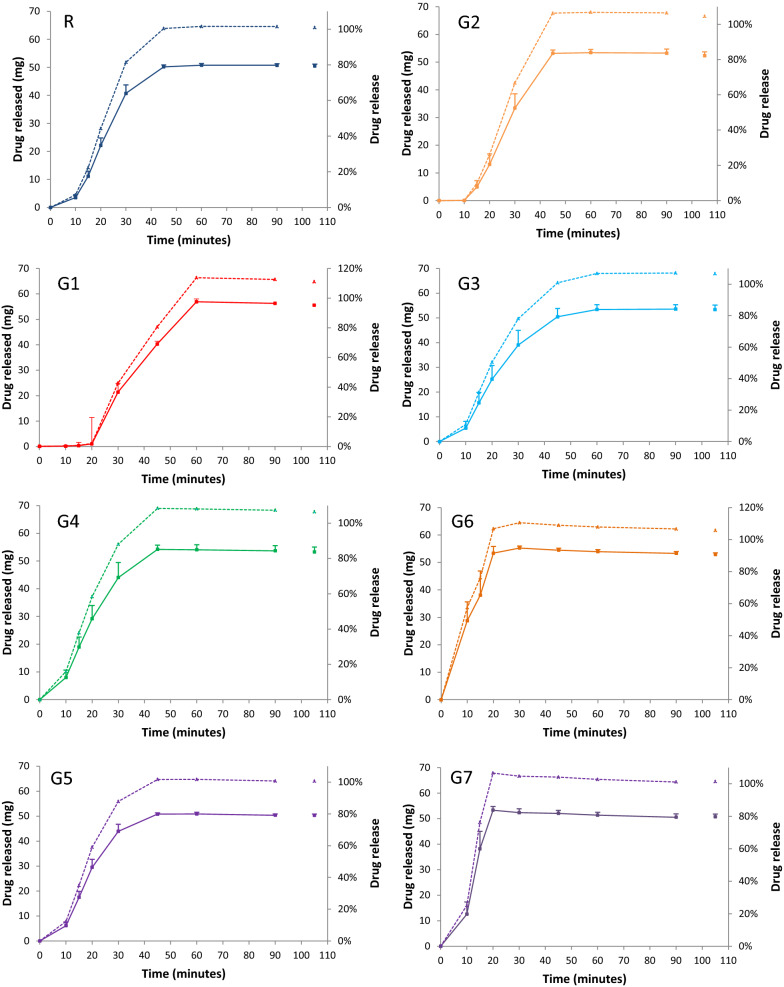
Dissolution profiles of a reference and seven generic enteric-coated 50 mg diclofenac sodium tablet formulations available on the Saudi market. R, reference, G1 to G7, generic formulations. Eight units of each formulation were studied. Mean (SD) amount of drug released at the specified times are shown on the left axis (continuous line) and percent of label amount released on the right axis (interrupted line). Time 0 min indicates amount released after 120 min in 0.1 N HCl. Other times indicate amount released in phosphate buffer (pH 6.8). Time 105 min indicates amount released with a stirring rate of 250 rpm for 15 min (infinity), otherwise, stirring rate was 50 ± 1 rpm, using USP dissolution apparatus type II (paddle apparatus) and a temperature of 37 ± 0.5 °C. Formulations’ label details are available in Additional file 1, Label information

Finally, despite having different dissolution profiles (Fig. 1), all eight formulations released on average ≥ 80% (Q (75%) + 5%) of their ASC within 45 min in phosphate buffer (pH 6.8). However, 3 of the 8 G1 unites released only 69%, 73%, and 75%, respectively. Time required to release 50% of ASC was about 21 min for the reference formulation and between 9 (G6) and 33 (G1) minutes for the generic formulations. No floating material, coning, gumming, capping or odd erosion pattern, sticking, air bubbles, or particles adhering to vessel or apparatus shaft was observed throughout the dissolution testing. In 0.1 N HCL for 2 h, G1 released 0.1% of its label ASC; the other formulations released 0.00%.

## Discussion

We evaluated in vitro quality of a reference and 7 generic formulations of 50 mg diclofenac sodium enteric-coated tablet that were commercially available on the Saudi market. Except for borderline performance of one generic formulation (G1), all formulations passed in vitro quality tests according to United States Pharmacopoeia [25]. Namely, weight variation of ≤ ±7.5% from mean weight for tablets ˃80 and < 250 mg and ≤ ±5% for tablets ≥ 250 mg; mean ASC between 90 and 110% of label; ≤ 1% friability weight loss; no disintegration in 0.1 N HCl (2 h) and complete disintegration in phosphate buffer within 60 min; and release of ≤ 10% of content in 0.1 HCl (2 h) and ≥ 80% within 45 min in phosphate buffer (each of 6 unites individually, or mean of 12 unites). Using stricter criteria, G1, G2, and G6 would fail the ASC test [26].

Our results on the reference formulation are consistent with the literature. Three studies that included the reference formulation (although from different manufacturing sites) found, respectively, that it has a mean weight of 216, 252, and 222 mg; ASC of 98.2%, 99.4%, and 99.3% compared to label; friability of 0.15%, 0.013%, and 0.82%; no disintegration in 0.1 N HCl, disintegration time of about 12, 22, and 51 min in phosphate buffer, and 50% content release in 20, 18.1, and not reported minutes [19–21].

As illustrated in case of G1, timely disintegration does not necessarily imply adequate dissolution. On the other hand, the disintegration apparatus mechanical force is probably lower than the destructive force of the gastrointestinal tract, so especially for enteric-coated products, potential in vivo disintegration in the stomach may not be necessarily detected in vitro. Because the typical paddle dissolution apparatus delivers even milder force, it is important to conduct disintegration testing despite having acceptable dissolution test results [27].

Although most commercially available generic formulations of diclofenac sodium tablets have been found to be of acceptable in vitro quality [19, 20, 22, 23], one of the three generic formulations in one study [19] inappropriately disintegrated in acidic medium, and 3 of four generic formulations in another study [21] released less than 60% at 45 min in phosphate buffer. In the current study, one of seven generic formulations had an ASC, friability, and hardness just beyond the acceptable limits and did not quite pass dissolution testing. The results indicate the importance of on-going surveillance of marketed formulations. In-vitro testing avoids exposing humans to drugs and is time and money saving, and has been proposed as replacement of in vivo bioequivalence testing under certain circumstances [28].

### Study strengths

This study is unique in examining a relatively large number of formulations, including a reference formulation, and using multiple-point dissolution curve rather than single points comparisons. It also used HPLC with the advantage of being able to separate diclofenac from potential interferences from formulation matrix/dissolution medium and detect drug degradation.

## Study limitations

We may have missed some diclofenac sodium 50 mg tablet formulations available on the Saudi market. Indeed, six formulations listed on the Saudi Formulary were not available in Riyadh pharmacies at the time of the study. However, since Riyadh is the capital of Saudi Arabia, we believe that we covered the most commonly used formulations. Our results do not apply to other diclofenac formulations (different strength, extended release) or to diclofenac potassium formulations. Finally, failing an in vitro test may be related to test performance rather than formulation performance, which is not likely in our study since reference formulation didn’t fail any test and several generic formulations passed.

## Supplementary information

**Additional file 1: Table S1:** Label information of a reference and seven generic enteric-coated 50 mg diclofenac sodium tablet formulations available on the Saudi market.

## Data Availability

Additional data are available in (Additional file 1: Table S1, Label information). Raw data are available from the corresponding author upon request.

## Abbreviations

ASC: Active substance content
G: Generic formulation
HCL: Hydrochloric acid
HPLC: High performance liquid chromatography
R: Reference formulation
SD: Standard deviation
