# Supplementary material for: Eight enteric-coated 50 mg diclofenac sodium tablet formulations marketed in Saudi Arabia: in vitro quality evaluation
Source: BMC Res Notes. 2020 Sep 14;13:428. doi: 10.1186/s13104-020-05270-4 (PMC7491129; doi:10.1186/s13104-020-05270-4)
Supplement: Supplementary file 1 — Additional file 1: Table S1: Label information of a reference and seven generic enteric-coated 50 mg diclofenac sodium tablet formulations available on the Saudi market. [file 13104_2020_5270_MOESM1_ESM.docx]

**Additional file-1, Label information**

**Table S1:** Label information of a reference and seven generic enteric-coated 50 mg diclofenac sodium tablet formulations available on the Saudi market

| **Code** | **Manufacturer** | **Manufacture date** | **Expiration date** | **Batch /lot number** | **Date Assessed** | **Active substance** | **Trade name** | **Form^1^** | **Dose**  **(mg)** |
| --- | --- | --- | --- | --- | --- | --- | --- | --- | --- |
| R | Novartis Pharma AG | 3/2019 | 2/2021 | KM068 | 1/2020 | Diclofenac sodium | Voltaren | Tablet | 50 |
| G1 | Jazzera pharma Ind. | 8/2018 | 8/2021 | 9205 | 1/2020 | Diclofenac sodium | Diclomax | Tablet | 50 |
| G2 | Hikma pharma | 7/2019 | 7/2021 | 7976A | 1/2020 | Diclofenac sodium | Votrex | Tablet | 50 |
| G3 | SPIMACO | 9/2018 | 9/2021 | 112068 | 1/2020 | Diclofenac sodium | Rofenac | Tablet | 50 |
| G4 | Riyadh Pharma | 11/2017 | 11/2020 | 17DR69 | 1/2020 | Diclofenac sodium | Rumafen | Gastro-resistant coated Tablet | 50 |
| G5 | Jamjoom Pharma Com | 1/2019 | 1/2021 | WA0037 | 1/2020 | Diclofenac sodium | Voltic | Coated tablet | 50 |
| G6 | Acino Pharma AG | 2/2018 | 2/2023 | 18043743 | 2/2020 | Diclofenac sodium | Olfen-50 | Film-coated tablet | 50 |
| G7 | Dar Al Dawa Dev & Inv. Co. LTD | 10/2019 | 10/2021 | 64FL | 2/2020 | Diclofenac sodium | Diclogesic | Coated tablet | 50 |

**^1^** Per Saudi Formulary. All formulations except G1 and G6 were labeled as enteric-coated in their inserts.
